# The benthic‐pelagic continuum: Age class and sex differences in the use of the vertical dimension by a rare pinniped

**DOI:** 10.1002/ece3.10601

**Published:** 2023-11-02

**Authors:** Mary‐Anne Lea, Lachlan W. Tainsh, Rob Mattlin, Leigh Torres, Kimberly Vinette Herrin, David R. Thompson, Mark A. Hindell

**Affiliations:** ^1^ Institute for Marine and Antarctic Studies University of Tasmania Hobart Tasmania Australia; ^2^ Marine Wildlife Research, Ltd Nelson New Zealand; ^3^ Department of Fisheries and Wildlife, Marine Mammal Institute, Hatfield Marine Science Center Oregon State University Newport Oregon USA; ^4^ Taronga Conservation Society Mosman New South Wales Australia; ^5^ NIWA Wellington New Zealand

**Keywords:** Campbell Island, diving, fisheries, foraging behaviour, Motu Ihupuku, satellite telemetry, sea lion, sub‐Antarctic, threatened species

## Abstract

Sea lions as a group, display strong site fidelity, and varying degrees of vulnerability to environmental change, disease and fisheries interactions. One of the rarest pinnipeds, the New Zealand sea lion (NZSL, *Phocarctos hookeri*) has a very restricted breeding range. At Campbell Island/Motu Ihupuku, one of the two primary breeding sites, at‐sea foraging behaviour is unknown. We hypothesised that NZSL of various sex and age classes would utilise the water column differently due to differing physiological constraints and therefore have different accessibility to prey resources. We tested whether sea lion diving behaviour varied in relation to (i) age and sex class, (ii) time of day and (iii) water depth. We also hypothesised that the proportion of benthic/pelagic diving, and consequently risk of fisheries interaction, would vary in relation to age and sex. Satellite telemetry tags were deployed on 25 NZSL from a range of age/sex classes recording dive depth, duration and location. Adult females and juveniles used inshore, benthic habitats, while sub‐adult males also utilised benthic habitats, they predominantly used pelagic habitat at greater distances from the island. Adult females and juveniles exhibited shorter dives than the same age/sex classes at the Auckland Islands, suggesting a lower dive effort for these age/sex classes at Campbell Island/Motu Ihupuku. Adult females dived more frequently than other age/sex classes, likely operating closer to their physiological limits; however, further data for this age class is needed. Sub‐adult male use of pelagic prey may increase their exposure to mid‐water trawls; however, further research detailing the degree of spatial overlap with fisheries is required. This study highlights the utility of spatially explicit dive data to predict vertical habitat use, niche separation of various age and sex classes of marine predators and attribute potential fisheries interaction risk in relation to predator habitat use.

## Graphical abstract

Sea lions as a group, display strong site fidelity, and varying degrees of vulnerability to environmental change, disease and fisheries interactions. We hypothesised that NZSL of various sex and age classes would utilise the water column differently due to differing physiological constraints and therefore have different accessibility to prey resources. We also hypothesised that the proportion of benthic/pelagic diving, and consequently risk of fisheries interaction, would vary in relation to age and sex. Adult females and juveniles used inshore, benthic habitats, while sub‐adult males also utilised benthic habitats, they predominantly used pelagic habitat at greater distances from the island. Adult females and juveniles exhibited shorter dives than the same age/sex classes at the Auckland Islands, suggesting a lower dive effort for these age/sex classes at Campbell Island/Motu Ihupuku. This study highlights the utility of spatially explicit dive data to predict vertical habitat use, niche separation of various age and sex classes of marine predators and attribute potential fisheries interaction risk in relation to predator habitat use.

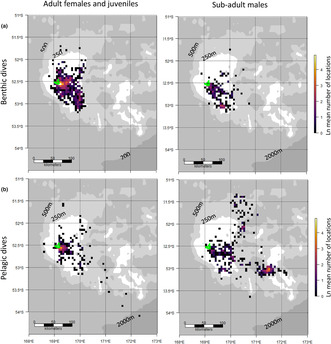

## 1 INTRODUCTION

Developing a thorough knowledge of marine predator foraging behaviour is integral to understanding the important role they play within their biological communities (Leung et al., 2013; Raymond et al., 2015; Roman et al., 2014) as integrators and indicators of ecosystem variability (Hazen et al., 2019). Knowledge of foraging behaviour (where, when and what individuals eat and how they search for prey) is required to determine how a species will be influenced by a changing environment. For air‐breathing marine predators, foraging behaviour is directly linked to physiological capacity, which is determined by the individual's available oxygen stores and the rate that these are consumed by metabolic processes (Costa et al., 2004). Diving performance for many pinnipeds increases with body mass, and therefore by age and sex, as males are generally larger than females (Weise & Costa, 2007). Age/sex classes with greater breath‐holding capacity can exploit habitat inaccessible to others, leading to reduced intraspecific competition (Sterling et al., 2014). Quantifying the variation in diving behaviour and habitat use between age and sex classes is necessary to more fully determine how the spectrum of individuals with a population might be influenced by both environmental changes and anthropogenic activities.

Sea lion species exhibit a variety of diving behaviours and exploit a range of habitats, but broadly, there are two typical foraging behaviours with contrasting population trends (Trites et al., 2006). Species which predominately prey on pelagic species, such as the Californian sea lion (*Zalophus californianus*), tend to have stable or increasing populations and exploit areas of higher marine productivity (2004). Conversely, species which exhibit regular benthic foraging, such as Australian (*Neophoca cinerea*) and New Zealand sea lions (*Phocarctos hookeri*), have strong breeding site fidelity and tend to have threatened populations in areas of low productivity (Campbell et al., 2006).

The New Zealand sea lion, the least populous sea lion in the world, is listed as *Nationally Vulnerable* within Aotearoa New Zealand (Baker, 2019) and was recently upgraded to *Endangered* by the International Union for the Conservation of Nature Red List (Chilvers, 2015). Studies on the sub‐Antarctic Auckland Islands, where ~68% of pup production is currently estimated to occur (Chilvers & Meyer, 2017) and the population has been declining since 1997/98 (Chilvers, 2015) have determined that New Zealand sea lions are the deepest and longest diving sea lion, they operate close to their physiological limits and forage primarily benthically (Chilvers & Wilkinson, 2009; Costa & Gales, 2000). Numbers of the second largest sub‐population of this species, at the more southerly Campbell Island/Motu Ihupuku, are thought to have increased in the last two decades, from an annual pup production of less than 50 to being now in excess of 500 (Childerhouse et al., 2005; Maloney et al., 2009), despite high levels of pre‐weaning pup mortality (Robertson & Chilvers, 2011) and considering differences in censusing methodologies (Maloney et al., 2009). Bycatch in the nearby southern blue whiting (*Micromesistius australis*) fishery has also reduced in recent years (Hamilton & Baker, 2019).

Comparison of these two main sub‐Antarctic populations, with documented contrasting population trends, is a unique opportunity to elucidate key underlying mechanisms of population regulation. At the Auckland Islands, several mechanisms have been postulated for a severe population decline that occurred from the late 1990s to late 2000s. These have included direct bycatch in the arrow squid (*Nototodarus sloanii*) trawl fishery operating around the Auckland Islands, indirect interactions with the fishery (nutritional stress and habitat degradation), climate variation and disease epizootics (Campbell et al., 2006; Roberts & Doonan, 2016; Robertson & Chilvers, 2011). Age‐specific differences have been linked to many of these mechanisms; in particular, adult females were identified as being vulnerable to direct mortality in the southern arrow squid fishery and juveniles are considered the most vulnerable to nutritional stress (Chilvers, 2008, 2012). However, management techniques implemented to mitigate the bycatch of sea lions in the squid trawl fishery have effectively reduced estimated bycatch to low levels (Hamilton & Baker, 2016).

We hypothesise that sea lions of various age and sex classes utilise the water column differently due to physiological constraints and associated accessibility of different prey resources. In this study, we aim to test whether the diving behaviour (depth, duration) of NZSL varies in relation to (i) age and sex class, (ii) time of day, (iii) site and (iv) habitat characteristics such as water depth.

## 2 MATERIALS AND METHODS

The study was conducted over 3 years (2012–2014) at Campbell Island/Motu Ihupuku (52°32′ S 169°8′ E), 644 km south of Aotearoa New Zealand. The most recent estimate (2009/10) of Campbell Island pup production was 681 pups, approximately 27% of the total species pup production (Maloney et al., 2009, 2012). In 2012 and 2013, juveniles and lactating adult female sea lions were captured manually at four different haul‐out sites in Perseverance Harbour (Table 1) using a specialised hoop net (Research Nets Inc. and Fuhrman Diversified Inc.) and then sedated using 2%–4% isoflurane in 4 L of oxygen. Isoflurane was administered by facemask using a closed circuit portable anaesthetic gas machine (Stinger, Advanced Anaesthesia Specialists; Gales & Mattlin, 1998). In 2014, lactating adult females and sub‐adult males were captured by remote injection using a Telinject rifle using 3 mL darts (Telinject Veterinarmedizinische Spezialgerate Gmbh). Sea lions were darted with Zoletil® 100 (Virbac (Australia) Pty. Ltd) at dose of 1.4–2.0 mg/kg (average of 1.8 mg/kg based on estimated body weight; Geschke & Chilvers, 2009) and supplemented with isoflurane in oxygen as above.

**TABLE 1:** Summary of dive behaviour data of the 21 *Phocarctos hookeri* tagged in this study relative to age and sex class.

Sea lion ID	Age class	Sex	Length (cm)	Deployment duration (day)	Depth mean (m)	Depth max. (m)	Duration mean (min)	No. of foraging trips	Mean trip duration (h at sea)	Mean ashore duration (h)
112686	Adult	F	173	70	88 ± 39	188	1.9 ± 0.7	10	25 ± 16	141.8 ± 361.1
112687	Adult	F	172	74	141 ± 62	325	2.5 ± 1.5	9	54.9 ± 19.8	123.4 ± 283.5
120484^a^	Adult	F	188	51	NA	NA	NA	10	87.3 ± 32.6	17.8 ± 11.5
120485^a^	Adult	F	185	187	NA	NA	NA	11	72.0 ± 12.2	21.2 ± 12.2
112688	Juv.	F	158	77	104 ± 4	225	2.3 ± 0.7	27	56.3 ± 55.6	14.8 ± 10.3
112689	Juv.	F	149	77	113 ± 5	325	2.2 ± 0.9	10	38.6 ± 27.7	150 ± 336
112690	Juv.	F	165	77	93 ± 5	188	2.1 ± 0.9	7	44.6 ± 58.3	64.5 ± 57
121809	Juv.	F	121	181	126 ± 4	188	2.4 ± 1.1	33	41.3 ± 18.2	70.2 ± 284.8
121810	Juv.	F	130	106	60 ± 5	325	2.2 ± 1.0	16	108.3 ± 101.1	125.3 ± 98.9
121812	Juv.	F	131	222	116 ± 6	275	2.1 ± 0.7	17	59.7 ± 48.2	820.2 ± 2359.3
121813	Juv.	F	151	22	132 ± 5	188	4.4 ± 2.5	9	31.7 ± 15.3	14.1 ± 10
121804	Juv.	M	147	12	131 ± 4	188	4.6 ± 2.2	4	46.1 ± 39.9	12 ± 9.4
121806	Juv.	M	132	102	136 ± 5	325	3.2 ± 1.3	13	140.7 ± 77.1	36.2 ± 22.5
121808	Juv.	M	144	182	106 ± 6	188	2.7 ± 0.9	12	81.6 ± 75.2	1171.2 ± 2535.4
121811	Juv.	M	115	165	59 ± 3	225	3.2 ± 1.7	40	75.6 ± 130.3	71.5 ± 137.4
137482	Juv.	M	168	141	50 ± 2	113	5.0 ± 1	13	194.1 ± 200.2	220.8 ± 209.9
138850	Juv.	M	158	228	97 ± 4	275	5.8 ± 0.8	18	140 ± 382.5	236.3 ± 816.3
137481	Sub‐adult	M	198	28	100 ± 4	163	6.1 ± 0.8	10	32.9 ± 24.1	35.7 ± 32.5
137483	Sub‐adult	M	188	102	112 ± 4	275	6.4 ± 1.9	15	110.7 ± 65.2	54.6 ± 25.6
137484	Sub‐adult	M	188	51	80 ± 42	225	4.9 ± 0.8	7	125.1 ± 72.1	42.4 ± 22.6
137485	Sub‐adult	M	185	187	93 ± 44	188	5.9 ± 1.2	17	71.6 ± 37.5	204.3 ± 419.7
137486	Sub‐adult	M	215	228	83 ± 51	275	5.3 ± 1.0	19	129.7 ± 72.1	149.6 ± 431.7
138851	Sub‐adult	M	178	94	114 ± 31	275	6.2 ± 0.8	17	64.2 ± 22.2	59.3 ± 90.8
Adult female (*N* = 2)			173 ± 0.7	72 ± 3	115 ± 38	256 ± 97	2.2 ± 0.5	9.5 ± 0.7	59.8 ± 26.7	76.0 ± 65.8
Juvenile female (*N* = 7)			144 ± 16	109 ± 69	104 ± 53	245 ± 63	2.3 ± 1.1	17 ± 10	37 ± 33	179.9 ± 286
Juvenile male (*N* = 6)			144 ± 19	138 ± 75	84 ± 50	219 ± 74	4.0 ± 1.7	17 ± 12	47 ± 56	291.3 ± 441.3
Sub‐adult male (*N* = 6)			192 ± 13	115 ± 78	70 ± 14	233 ± 50	5.8 ± 0.6	14.1 ± 4.7	84 ± 59	91 ± 69.3

*Note*: Values presented are means and standard deviation.

^a^ Seals without dive data and so not included in dive statistics.

The sea lions were equipped with satellite‐linked SPLASH tags (Wildlife Computers), 138 × 38 × 20 mm, weight 145 g, which estimated locations using Argos satellites, and transmitted dive depth (±0.5 m) and duration (s). The tags were attached onto the fur on the dorsal midline of the sea lion, posterior to the scapulae, with a fine layer of epoxy. Dive depth and duration information were recorded each second during dives. Each individual dive was binned into 14 user‐defined data ranges over 6 h summary periods prior to transmission, producing four summary histograms daily. The histogram periods ranged from, 1:00 to 6:59:59, 7:00 to 12:59:59, 13:00 to 18:59:59, 19:00 to 0:59:59 GMT.

As the age of the individual NZSL was unknown, each sea lion was allocated to an age and sex class based on their standard length: Females with standard lengths greater than or equal to 1.7 m were classified as adult, males greater than or equal to 1.7 m were classified as sub‐adult (as no adult males were captured; Childerhouse et al., 2010). All animals less than 1.7 m were classified as juveniles.

Data were extracted using the WC‐DAP program (Wildlife Computers Data Analysis Programs, V:3.0.326.0 09). Dives <10 m were not recorded for the 2014 cohort, and so these shallow dives were also excluded from the 2012 and 2013 data sets. Dives with durations <20 s were also discarded, as dives within these ranges were transiting or reflecting other surface behaviours.

We used a Kalman filter (*Crawl* package in R, Johnson, 2016; Johnson et al., 2008) to obtain the best, estimated path of each sea lion, using 2‐h time steps. One location every 2 h corresponded to the overall mean daily rate of location estimates (12 per day) provided by Argos.

Mean dive depth (m) and dive duration (s) and the proportion of dives within each depth/duration bin were estimated for each 6‐hourly period following (Lea et al., 2010). The filtered tracks provided three estimated locations for each 6‐hourly period, and for each of these the distance from Campbell Island was calculated, as was the bathymetry for each location. Bathymetry data were extracted from the National Oceanic and Atmospheric Administration (http://www.ngdc.noaa.gov/mgg/bathymetry/multibeam.html). Each 6‐hourly period was then categorised as *inshore* when maximum bathymetry was ≤200 m and *offshore* when maximum bathymetry >200 m.

The durations of foraging trips and haul‐outs were calculated using a threshold distance of 1 km from the Campbell Island coast to denote the start and end of a foraging trip. This threshold allowed for the inherent uncertainty in the Argos location estimate and ensured that only trips further than this threshold were included in the subsequent analyses.

The proportion of benthic dives in each 6‐hourly period was calculated. This was done by using 80% of the shallowest of the three bathymetry records obtained during a 6‐hourly histogram as a depth threshold. All dives in bins deeper than this threshold were classified as benthic. The depth threshold was deliberately conservative to ensure that no underestimates of benthic foraging occurred due to inherent uncertainties in Argos‐derived sea lion locations (Vincent et al., 2002).

The proportion of 6‐hourly periods occurring within daylight hours was also estimated. Nautical dawn and dusk (solar elevation equal to 12 u below horizon) were calculated using each seal's estimated latitude and longitude at the start and end of a histogram period (see Sterling et al., 2014). *Proportion daylight* was estimated as the amount of the 6‐hourly histogram within daylight hours. *Proportion daylight* was then used to categorise diving activity as occurring either during the day (daylight proportion = 1.0), during the night (daylight proportion = 0) or during crepuscular periods (dives ≠1 or ≠0).

Linear mixed‐effects models were used to explore the relationship between continuous dive behaviours (depth, duration and benthic diving) and explanatory variables (age and sex class, proportion daylight) with individual sea lion ID as random term using the nlme package (Pinheiro et al., 2016) in (R Core Team, 2013). Model selection was performed by ranking models in increasing order of AIC (Akaike Information Criterion). *Age* and *sex classes* are categorical variables and *proportion daylight* is continuous. Visual inspection of response variables showed that log‐transformation dive depth and duration parameters was not necessary.

We compared the dive depths and durations of adult females and juvenile New Zealand sea lions from Campbell Island/Motu Ihupuku with published values reported for two other colonies (1): the Auckland Islands and the Otago Peninsula on mainland New Zealand Individual values for mean dive depth (m) and dive duration (s) for adult females and juveniles at the three locations were compared using linear models. Response variables were log transformed where necessary. Only data for adult females and juveniles (defined as less than 5 years of age (Auckland Islands and Otago Peninsula) or ≤1.7 m in body length (this study)) were available from all three sites (see 1 for data used in analyses). We also only used studies which used 6 m as the minimum dive depth to ensure comparability among the data sets, excepting individuals from the Otago Peninsula population where a dive depth threshold of 3 m was applied (Augé, Chilvers, Davis, & Moore, 2011; Augé, Chilvers, Moore, & Davis, 2011).

## 3 RESULTS

**FIGURE 1:**
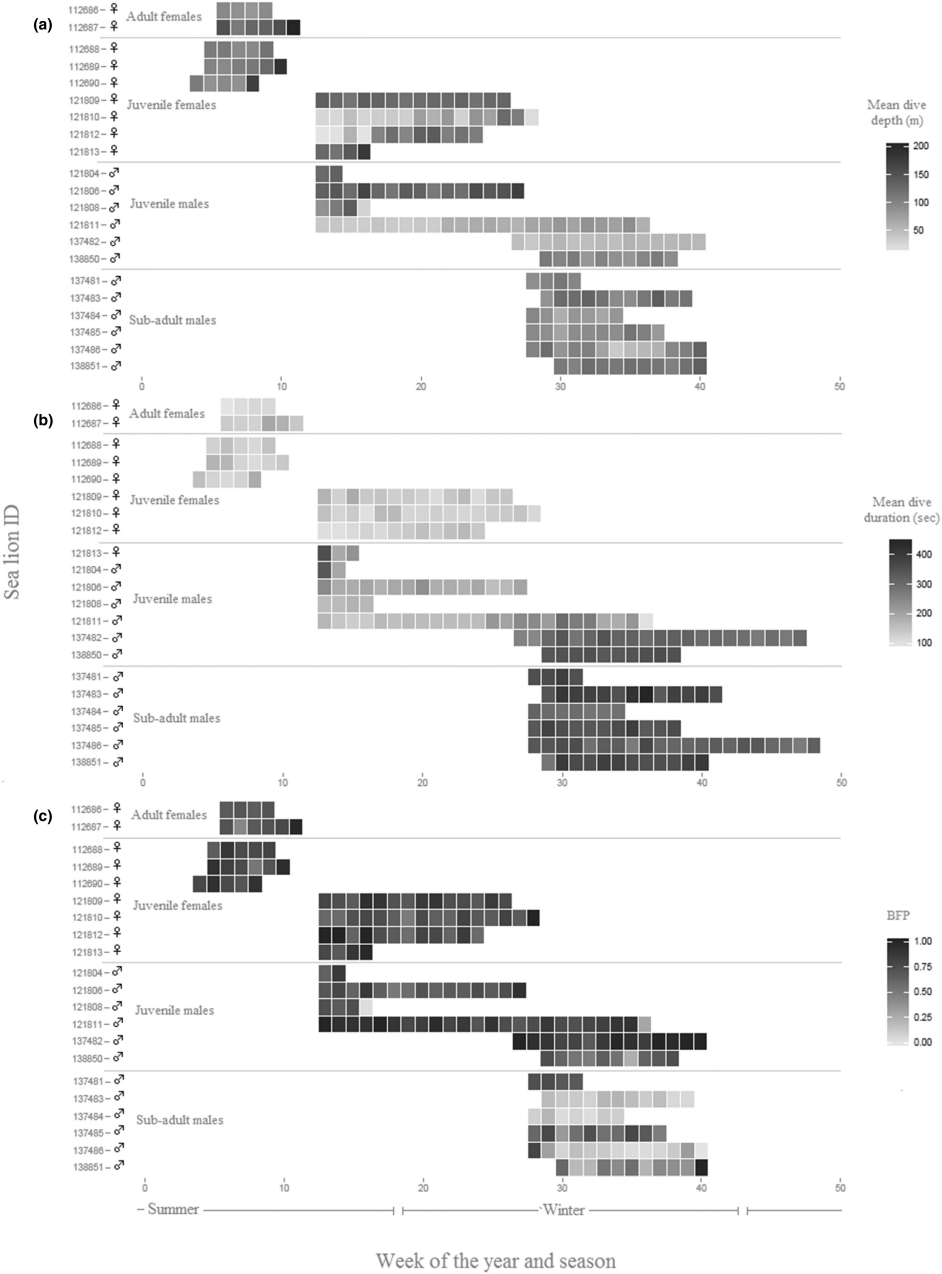
Weekly means plot of (a) dive depth, (b) dive duration and (c) proportion of benthic foraging, by age class and sex of 21 *Phocarctos hookeri* at Campbell Island/Motu Ihupuku.

More than 3 days of data were collected for 21 of the 25 deployments (Table 1), providing 5158 6‐h periods from 1282 days of data (Figure 1, Table 2). Location data were recorded for an additional two *adult females* although no dive data were available for these individuals and therefore were not included in the dive analysis. The dataset encompasses movement and diving information for male and female juvenile NZSL and sub‐adult male and adult female NZSL (Table 1) over 3 years and across seasons (Figure 1).

**TABLE 2:** Deployment information of 21 *Phocarctos hookeri*.

Seal lion ID	Year	Age Class	Number of Histograms at Different Times of the day			Summer		Autumn			Winter			Spring			
			Day Dives	Crepuscular Dives	Night Dives	Jan.	Feb.	Mar.	Apr.	May	Jun.	Jul.	Aug.	Sep.	Oct.	Nov.	Dec.
112686	2012	Adult female	23	25	0												
112687	2012	Adult female	34	41	0												
112688	2012	Juvenile female	30	36	0												
112689	2012	Juvenile female	38	36	0												
112690	2012	Juvenile female	33	20	0												
121809	2013	Juvenile female	99	0	93												
121810	2013	Juvenile female	96	0	103												
121812	2013	Juvenile female	63	0	58												
121813	2013	Juvenile female	19	0	13												
121804	2013	Juvenile male	8	0	11												
121806	2013	Juvenile male	110	0	115												
121808	2013	Juvenile male	32	0	35												
121811	2013	Juvenile male	217	2	210												
137482	2014	Juvenile male	165	107	45												
138850	2014	Juvenile male	48	37	46												
137481	2014	Sub‐adult male	22	13	10												
137483	2014	Sub‐adult male	61	57	47												
137484	2014	Sub‐adult male	35	26	35												
137485	2014	Sub‐adult male	43	52	46												
137486	2014	Sub‐adult male	136	76	53												
138851	2014	Sub‐adult male	55	39	44												

*Note*: The number of 6‐h histograms occurring during different times of the day and the deployment periods of SPLASH tags throughout the year. Shading indicates the timing of deployment for individual New Zealand sea lions.

### 3.1 Foraging trip and haul‐out durations

A total of 344 complete foraging trips were recorded with an average of 15.0 ± 8.5 trips per sea lion and lasting a mean of 115.5 ± 68.6 days in duration.

The duration of foraging trips and haul‐out periods varied between sex/age classes (Table 1). *Sub‐adult males* made the longest trips (84 ± 59 h) and *juvenile females* made the shortest (37 ± 33 h) trips. *Adult* (lactating) *females* had the shortest haul‐out periods (76.0 ± 65.8 h), while *juvenile males* had the longest haul‐out durations (291.3 ± 441.3 h).

### 3.2 Dive depth and duration

**FIGURE 2:**
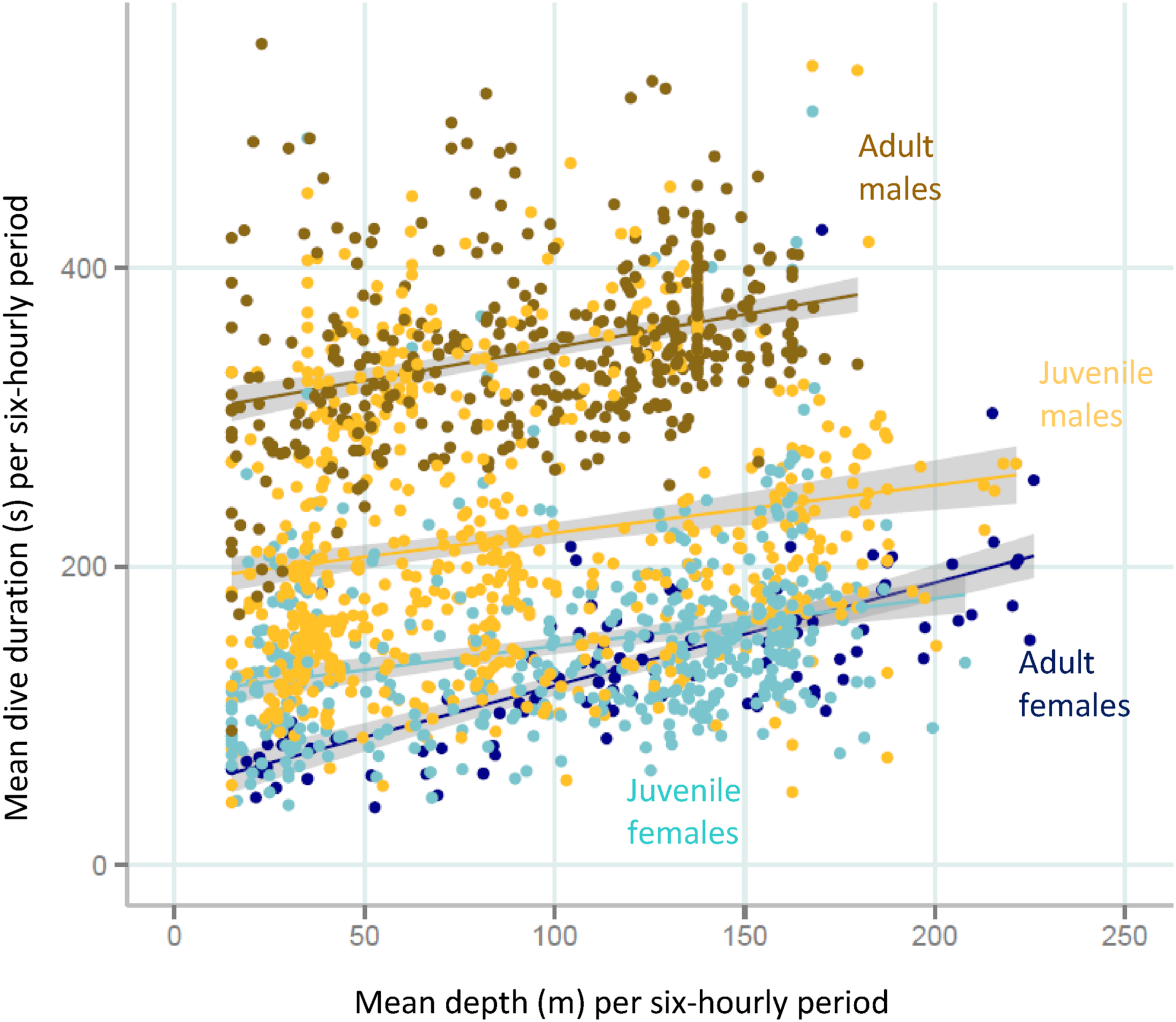
Relationship between mean dive depth and mean dive duration of 6‐hourly histograms (*n* = 1696), for the sex and age classes of New Zealand sea lions at Campbell Island/Motu Ihupuku. The solid lines represent the lines of best fit derived from a linear regression and the shaded regions indicated the 95% confidence intervals of those models.

**FIGURE 3:**
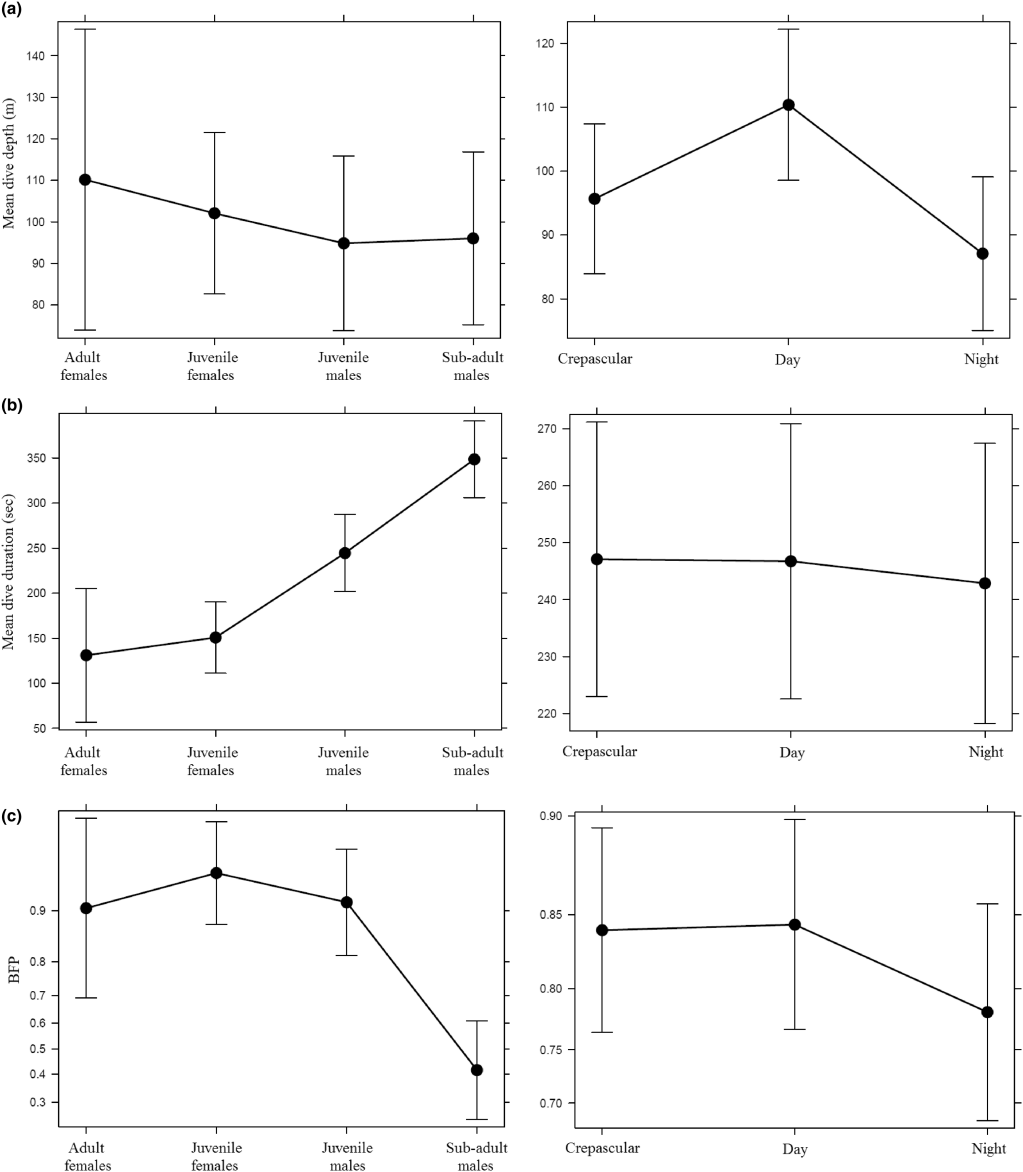
Plots of linear mixed‐effects models fit by restricted maximum likelihood for (a) mean dive depth, age class and time of day, (b) mean dive duration, age class and time of day and (c) a generalised linear mixed model fit by maximum likelihood plot of benthic foraging, age class and time of day for NZSL at Campbell Island/Motu Ihupuku. BFP, proportion of benthic foraging.

Dive depth was positively related to dive duration in all age classes (Figure 2). *Adult females* dived deeper than the other sex/age classes with a mean of 114.6 ± 37.6 m; *sub‐adult males* made the shallowest dives (70.0 ± 14.4 m, Table 1, Figure 1). The best model explaining dive depth included both *age class* and *time of day*, although of these, *time of day* was the most influential variable (Table 3). The shallower dives occurred during the night and crepuscular periods (Figure 3a, Table 3). It was not possible to investigate the interaction between *age class* and *time of day* because, not all age classes made dives for each time of day.

**TABLE 3:** Dive behaviour linear mixed‐effects models with response variables dive depth and dive duration, using the response variables age class, sex and diurnal.

Dive parameter	Predictor variable	*k*	AIC	ΔAIC	wAIC
Dive depth (m)	Diurnal + Age class	5	23,364	0	1
	Diurnal	4	23,381	16	0
	Age class	4	23,454	91	0
	Null	3	23,471	108	0
Dive duration (min)	Diurnal + Age class	5	33,228	0	0.95
	Age class	4	33,233	6	0.5
	Diurnal	4	33,276	48	0
	Null	3	33,281	54	0
Benthic foraging	Diurnal + Age class	5	1725	0	83
	Age class	4	1729	3	17
	Diurnal	4	1740	15	0
	Null	3	1743	18	0

*Note*: Models are ranked by AIC with ΔAIC = 0 denoting the best model.

Abbreviations: AIC *k*, the number of terms in the model; AIC, Akaike information criterion (AIC) a measure of the relative quality of the statistical model; wAIC, weighted AIC; ΔAIC, the delta AIC (the difference in AIC between sequential models).

Variation in dive duration was best explained by both *age sex class* and *daynight* (Table 3 and Figure 3b). In this case, *age sex class* was the most influential variable; including the *diurnal* term improved the model only moderately (ΔAIC = 3) suggesting that it was relatively weak effect. S*ub‐adult males* had the longest dives (5.8 ± 0.6 min), followed by *juvenile males* (4.0 ± 1.7 min), then *juvenile females* and *adult females* (2.3 ± 1.1 and 2.2 ± 0.5 min, respectively).

### 3.3 Benthic and pelagic foraging behaviour

Variation in the proportion of benthic dives in a 6‐hourly period was best explained by both *age class* and *time of day* (Table 3). *Sub‐adult males* exhibited a lower proportion of benthic dives (0.36 ± 0.26) than the other age classes (Figure 3c, Table 4). There was also a lower proportion of benthic dives at night‐time overall, although this effect was relatively weak (Figure 3c). *Sub‐adult males* also foraged further from Campbell Island (101 ± 61 km), and a lower proportion of their dives occurred in inshore waters (0.56 ± 0.40, Table 4). *Juvenile males*, *juvenile females* and *adult females*, on the other hand, made a similar proportion of benthic dives (0.70 to 0.78), inshore dives (0.87 to 0.98) and foraged, on average, within 45 km of the island (Table 4).

**TABLE 4:** Summary of foraging type and habitats of the 21 *Phocarctos hookeri*.

Sea lion ID	Age class	Sex	Proportion of inshore dives	Max distance from CI (km)	BFP
112686	Adult	F	1.00	17 ± 12	0.68 ± 0.02
112687	Adult	F	0.73	68 ± 31	0.71 ± 0.17
112688	Juvenile	F	1.00	21 ± 16	0.77 ± 0.08
112689	Juvenile	F	1.00	17 ± 16	0.77 ± 0.16
112690	Juvenile	F	1.00	6 ± 4	0.82 ± 0.10
121809	Juvenile	F	0.97	24 ± 11	0.78 ± 0.09
121810	Juvenile	F	0.93	14 ± 20	0.70 ± 0.12
121812	Juvenile	F	0.96	36 ± 26	0.77 ± 0.17
121813	Juvenile	F	1.00	14 ± 5	0.83 ± 0.13
121804	Juvenile	M	1.00	43 ± 21	0.75 ± 0.16
121806	Juvenile	M	0.85	71 ± 25	0.69 ± 0.11
121808	Juvenile	M	0.88	59 ± 7	0.54 ± 0.34
121811	Juvenile	M	1.00	5 ± 7	0.82 ± 0.15
137482	Juvenile	M	1.00	3 ± 8	0.90 ± 0.10
138850	Juvenile	M	0.88	52 ± 61	0.59 ± 0.15
137481	Sub‐adult	M	1.00	27 ± 25	0.71 ± 0.04
137483	Sub‐adult	M	0.19	167 ± 58	0.13 ± 0.06
137484	Sub‐adult	M	0.18	166 ± 57	0.09 ± 0.06
137485	Sub‐adult	M	0.99	58 ± 27	0.60 ± 0.14
137486	Sub‐adult	M	0.23	128 ± 61	0.17 ± 0.21
138851	Sub‐adult	M	0.74	59 ± 46	0.44 ± 0.23
Adult female (*n* = 2)			0.87 ± 0.19	44 ± 36	0.70 ± 0.02
Juvenile female (*n* = 7)			0.98 ± 0.03	21 ± 18	0.78 ± 0.04
Juvenile male (*n* = 6)			0.93 ± 0.07	29 ± 39	0.72 ± 0.14
Sub‐adult male (*n* = 6)			0.56 ± 0.40	101 ± 61	0.36 ± 0.26

*Note*: Habitat is described as the proportion of inshore versus offshore dives, where inshore is defined as being within the 200 m bathy metric contour. Also listed is the mean maximum distance from Campbell Island/Motu Ihupuku (CI) that a sea lion reached across all foraging trips. Also indicated is the proportion of dives that were classified as benthic foraging proportion (BFP), where a benthic dive is define as being deeper than the 80 percentile of ocean depths in a 6‐h period. Values presented are means and standard deviation.

**FIGURE 4:**
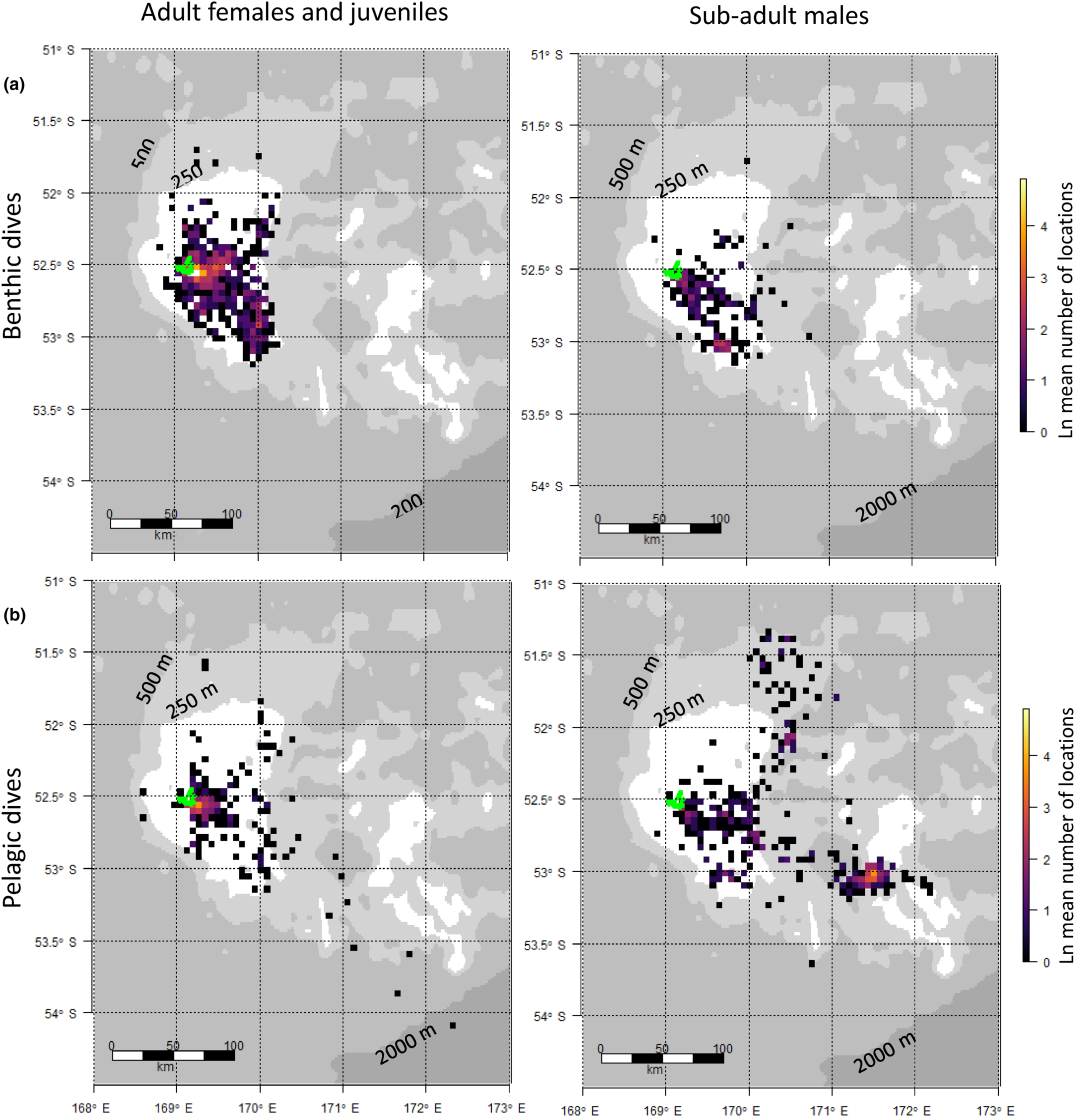
Distributions of locations of either (a) benthic or (b) pelagic dives. *Adult* and *juvenile females* along with *juvenile males* are grouped due to their similar foraging characteristics (i.e. all are inshore and benthic). Plotted are the mean number of locations that the individual seals spent in each 5 × 5 km grid cell. Bathymetry is demarcated by grey colouring with numbers indicating depth and the edge of the Campbell Plateau expressed in dark grey.

**FIGURE 5:**
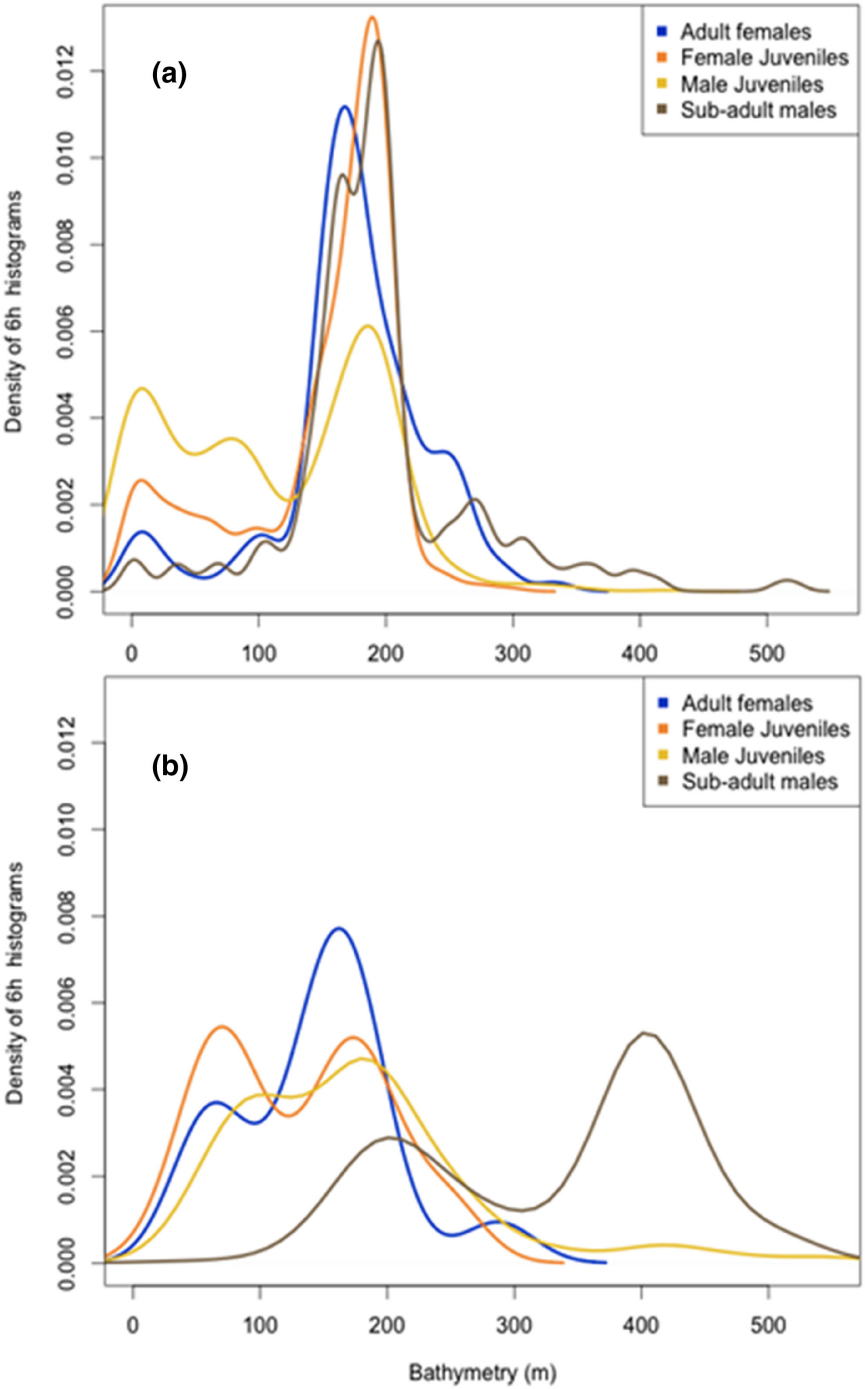
Density plot of bathymetry for all locations of sea lions at Campbell Island/Motu Ihupuku associated with (a) benthic or (b) pelagic 6‐hourly dive histograms.

Benthic diving was largely restricted to waters less than 250 m (Figure 4), while pelagic dives were more widespread across depth ranges (Figures 1 and 3). Benthic dives for all sex classes occurred over the relatively narrow bathymetry depth range of 150–200 m, with very few benthic dives in depths >200 m (Figure 5a). The depths of *adult female* and *juvenile female* pelagic dives were more variable, although again peaks occur over the 150–200 m ocean depths. *Sub‐adult male* pelagic foraging had a bimodal distribution, with an inshore mode occurring over depths of 200 m and a second mode centred over depths of 400 m (Figure 5a,b).

### 3.4 Comparisons among sites

**FIGURE 6:**
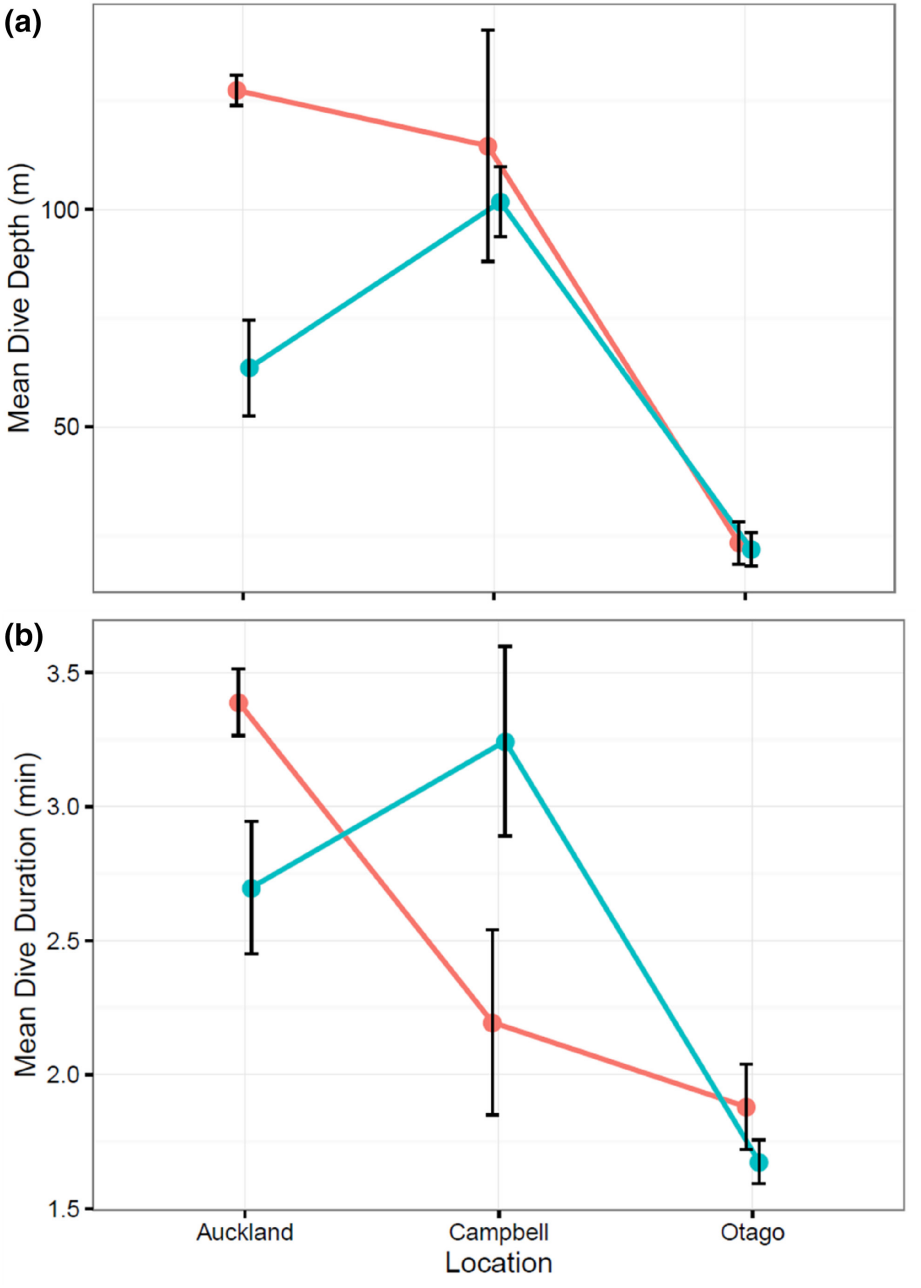
Comparisons of adult female and juvenile NZSL from the Auckland Islands, the Otago Peninsula and Campbell Island/Motu Ihupuku for (a) mean dive depth ± SE and (b) mean dive duration ± SE. Summary values for the Auckland Islands individuals were extracted from (see Table S1).

We compiled diving data from 107 individual sea lions from the three sites: Campbell Island/Motu Ihupuku, Auckland Islands and the Otago Peninsula on Aoetearoa New Zealand's South Island (Table S1). Diving behaviour of adult females has been quantified at two sites (Enderby and Dundas Islands) and was not significantly different between the sites (Chilvers et al., 2020). We therefore used the data from Enderby Island, as it represented all the sex and age classes, for our inter‐island comparisons. The number of adult females was small for both Campbell Island/Motu Ihupuku and Otago Peninsula (two and five, respectively), so caution is required interpreting this aspect of the analyses. For dive depth, both age (*F*_75,1_ = 50.2, *p* < .001) and location (*F*_75,2_ = 40.5, *p* < .001) were important explanatory variables, as was their interaction (*F*_75,2_ = 6.2, *p* < .01). Female sea lions at the Auckland Islands made deeper dives on average than juveniles at that site (126 ± 29 and 62 ± 42 m, respectively, Figure 6a). However, at Campbell Island/Motu Ihupuku adult females and juveniles dived to similar depths. The two adult females from Campbell Island/Motu Ihupuku dived to similar depths to those recorded for females from the Auckland Islands. Adult females and juveniles from the Otago Peninsula made shallower dives than at the other sites (23 ± 10 and 21 ± 10 m, respectively, Figure 6a).

For dive duration, only location (*F*_75,2_ = 8.7, *p* < .01) was an important explanatory variable. Overall, the Auckland Islands animals dived longest, followed by those from Campbell Island/Motu Ihupuku, with the Otago Peninsula sea lions recording the shortest dives (Figure 6b).

## 4 DISCUSSION

Our study reports on the diving behaviour of a rare and endangered pinniped at a significant breeding site, for which foraging behaviour was previously unknown. The diving behaviour of vertebrate marine predators provides a variety of metrics relevant to their physiology, ecology and surrounding environment (Aguilar Soto et al., 2008; Bost et al., 2015; Viviant et al., 2014; Womble et al., 2013). In this study, the diving behaviour and foraging habitats of New Zealand sea lions from Campbell Island/Motu Ihupuku were influenced by age and sex. The larger *sub‐adult males* made longer foraging trips, travelled further, and made more offshore and pelagic dives than the *juveniles* and *adult females*. They also dived for almost twice as long *adult* and *juvenile females*. Pup‐rearing obligations of *adult females* likely restricts their foraging range to shorter distances from Campbell Island/Motu Ihupuku, while physiological capabilities and social determinants, such as the need to develop social behaviours on land (Gentry, 1974), likely limit the foraging range and behaviour of *juveniles*. We presented diving data for sub‐adult male NZSL (≥1.7 m), an age/sex class which has previous been unstudied for this species and which has known bycatch interactions with sub‐Antarctic fisheries (Abraham & Berkenbusch, 2017; Hamilton & Baker, 2019).

Larger and older diving predators, including sea lions, typically have superior diving capabilities due to greater oxygen stores, more developed physiological control and morphological development (Butler & Jones, 1997; Costa et al., 2004; Fowler et al., 2006; Thompson & Abraham, 2010; Weise & Costa, 2007). Oxygen storage, blubber development for insulation and development of energy‐efficient methods of locomotion are all important aspects of ontogenetic development (Costa & Gales, 2000; Prewitt et al., 2010). Therefore, older age classes have the potential to reach and exploit habitat largely inaccessible to *juveniles*, both in terms of geographic distance and position in the water column (Fowler et al., 2006, 2007).

Our results were broadly consistent with this pattern; the largest age class, *sub‐adult males*, undertook the longest dives (mean 5.8 min), followed by *juvenile males* (4.0 min), and *juvenile* and *adult females*; all of which displayed similarly short dives (2.2–2.3 min on average). Depths attained by the various age classes, however, were reasonably similar, with a slight tendency for the *juveniles* to dive deeper than the *sub‐adult males*. The relatively short, deep dives of the *juveniles* and *females* indicate that they would have to either swim more quickly, or dive more frequently, to achieve the same net time in the foraging zone (bottom of dive) as *sub‐adult males*. By predominantly diving benthically (~80% of all dives) and inshore, *juveniles* are employing a strategy that is inherently costly. Conversely, *sub‐adult males*, while having the body size necessary to undertake deeper benthic dives, spent ~60% of their time in relatively shallow (~70 m) pelagic dives. Body size and oxygen stores are therefore not the only determinants of dive duration, but ecological factors are also important.

Sex class is another important determinant of diving behaviour, as demonstrated by the differences between *juvenile males* and *females* at Campbell Island/Motu Ihupuku. Despite both *juvenile males* and *juvenile females* being predominantly benthic foragers and of similar body size, males dived for significantly longer than females during dives to similar depths. Adult sea lions are highly sexually dimorphic both in size and in terms of foraging behaviour (Lindenfors et al., 2002). This early differentiation in diving behaviour at Campbell Island, and the Auckland Islands (Leung et al., 2012, 2014), may indicate that differences in diving ability between *males* and *females* start to commence in advance of divergence in body size as appears to be the case in Antarctic fur seals (*Arctocephalus gazella*, Kernaléguen et al., 2016).

Benthic foraging for all age classes took place typically near Campbell Island over a bathymetric range of 150–200 m, although *adult females* and *juveniles* of both sexes exhibited little pelagic foraging. All the foraging effort for these sea lions was predominately to the east of Campbell Island over the shelf. At the Auckland Islands, foraging behaviour for these age classes is also largely confined to the shelf (Leung et al., 2012). In contrast, *sub‐adult males* at Campbell Island/Motu Ihupuku, made a significantly greater proportion of pelagic dives, many over deeper waters >400 m. It has been shown that sea lion species with a predominantly pelagic foraging strategy operate well within their calculated aerobic dive limit and expend less energy, spending more time at the surface resting (Costa et al., 2004). The limited pelagic diving by *adult females*, albeit a small sample size, and *juveniles* in this study, suggest that they target benthic prey species, even though this may require more energy. Benthic prey, although more sparsely distributed, tend to be larger and more predictable than pelagic prey, which may help support dependent pups (Costa & Gales, 2003). Alternatively, pelagic prey may be less abundant in coastal waters. This is indicated by the location of commercial fisheries targeting the pelagic southern blue whiting which operate in waters more than 400 m depth around Campbell Island/Motu Ihupuku (Cole et al., 2013) and report interactions with largely male New Zealand sea lions (Thompson et al., 2015). The constraint of returning regularly to shore, restricting suckling mothers to short foraging trips, may mean that *adult females* are confined to a restricted range of ocean depths with little access to seasonal high‐energy pelagic prey such as southern blue whiting. Although benthic prey species are influenced less by seasonal fluctuations, the seafloor habitat is more susceptible to degradation from mid and bottom trawls, such as the ling fishery that operates in waters surrounding Campbell Island/Motu Ihupuku (see MPI, 2017). The degree of competition between fisheries and Campbell Island *female* sea lions is difficult to determine without first quantifying the degree of overlap between New Zealand sea lions and fisheries effort.

Contrasting foraging behaviour within a species is not uncommon and has been linked to habitat in Californian sea lions (Costa et al., 2004), seasonal changes in productivity in Galapagos sea lions (*Zalophus wollebaeki*; Villegas‐Amtmann et al., 2008) and niche divergence between sexes in New Zealand sea lions (Leung et al., 2012). At Campbell Island/Motu Ihupuku, clear differences in horizontal and vertical habitat use were detected between various age and sex classes. Habitat used by *sub‐adult males* for pelagic diving was bimodal, with a shallow mode near Campbell Island/Motu Ihupuku and a second over deeper waters (400 m) further from shore. Benthic dives for this age class, however, occurred almost exclusively in water depths of ~110–210 m. This diversity in foraging habitat used by *sub‐adult males* is likely a product of their longer and more distant foraging trips, made possible by males not needing to return to land as frequently as lactating females. Conversely, *juvenile* sea lions of both genders also made relatively short trips and performed predominantly benthic dives. These animals are the smallest of those studied and therefore have the most restricted dive capabilities. Yet they are not constrained by suckling requirements, so it is less clear why they remain in coastal waters and exploit the less densely aggregated benthic prey (Costa et al., 2004). There may be other social determinants requiring them to spend more time on land than older males, as well as other deterrents to foraging further afield on more profitable pelagic prey, such as predation risk (Wirsing et al., 2008) or lack of experience in finding and handling different prey types due to their age.

Despite the relatively small numbers of foraging trips recorded at Campbell Island/Motu Ihupuku and the staggered deployments across age/sex classes and years/season in this study, we can make cautious comparisons between the diving behaviour of *adult female* and *juvenile* sea lions (males and females combined) there with those on the Auckland Islands and at the Otago Peninsula on New Zealand's South Island (Augé, Chilvers, Davis, & Moore, 2011; Augé, Chilvers, Moore, & Davis, 2011; Figure 6). At the Auckland Islands, *adult females* made longer dives but to similar depths than the two mature females from Campbell Island/Motu Ihupuku that were followed during the winter months in this study (Figure 6). This suggests that the Auckland animals have higher foraging effort potentially due to lower prey densities linked to intraspecific competition or inter‐annual variability in the prey field. This offers limited support for the hypothesis that sea lions from the Auckland Islands, are subject to nutritional stress as discussed in Chilvers et al. (2006), although the determinant of this stress is uncertain. The comparisons with seal lion diving behaviour at the Otago Peninsula are likely influenced by the discrepancies in dive definitions for the different sites. At the Otago Peninsula dives were classified as >3 m depth and almost 50% of dives recorded were between 3 and 10 m depth. At other sites dives were classified as > or ≥6 m. Taking these differences into account it is still likely that the shorter, shallower dives exhibited by *adult females* recolonising the Otago Peninsula, particularly when coupled with their pelagic diet and high foraging site fidelity (Augé et al., 2014), confirm the hypothesis that these sea lions can more easily access productive habitat than sea lions breeding in sub‐Antarctic latitudes. Historically, New Zealand sea lions inhabited the North and South islands of New Zealand in significant numbers until they were virtually extirpated though human hunting prior to European colonisation (Augé, Chilvers, Moore, & Davis, 2011; Childerhouse & Gales, 1998). The colony at Campbell Island is at the southern extent of the species' range, and it therefore seems more than likely that they are living and foraging at the limits of their capacity at this site.

## 5 CONCLUSIONS

These preliminary findings have permitted an insight into the seasonal foraging behaviours of New Zealand sea lions at this most southerly breeding site. Using summarised, spatially explicit satellite‐transmitted data on diving behaviour we have shown that there is considerable variation in dive behaviour between age and sex classes at Campbell Island/Motu Ihupuku, and in comparison with New Zealand sea lions at other breeding sites. This variability is likely due to age and sex‐related physiological constraints, breeding requirements, time of year, prey distribution and likely habitat quality. Further to this, intraspecific competition in other benthically feeding pinniped species, such as the grey seal (*Halichoerus grypus*), has been linked to population trajectories (Breed et al., 2013). The potential for and influences of such competition requires further investigation should the population continue to increase at Campbell Island/Motu Ihupuku. Similarly, simultaneous deployments across all age/sex classes in this study would help to elucidate the seasonal component of behavioural dynamics. Additionally, the diving behaviour of newly weaned pups and lactating adult female NZSL requires greater understanding at this site to more fully assess how changes in dive behaviour are linked to prey fluctuations, intraspecific competition and the trajectory of the Campbell Island/Motu Ihupuku population.

Finally, this study highlights the broader utility of using spatially explicit dive parameters to predict vertical habitat use, niche separation in various age and sex classes and attribute potential fisheries interaction risk in relation to water column usage for rare, vertebrate marine predators.

## AUTHOR CONTRIBUTIONS

**Mary‐Anne Lea:** Conceptualization (equal); data curation (equal); formal analysis (lead); investigation (equal); methodology (equal); project administration (equal); supervision (equal); validation (equal); visualization (equal); writing – original draft (lead); writing – review and editing (lead). **Lachlan W. Tainsh:** Data curation (supporting); formal analysis (equal); methodology (equal); visualization (supporting); writing – original draft (lead); writing – review and editing (supporting). **Rob Mattlin:** Conceptualization (equal); data curation (equal); funding acquisition (lead); investigation (equal); methodology (equal); project administration (equal); writing – original draft (supporting). **Leigh Torres:** Conceptualization (equal); methodology (equal); writing – original draft (equal); writing – review and editing (equal). **Kimberly Vinette Herrin:** Investigation (equal); methodology (equal); writing – original draft (equal); writing – review and editing (equal). **David R. Thompson:** Conceptualization (equal); funding acquisition (equal); methodology (equal); project administration (equal); writing – original draft (equal); writing – review and editing (equal). **Mark A. Hindell:** Conceptualization (equal); data curation (equal); formal analysis (lead); investigation (equal); methodology (equal); project administration (equal); supervision (equal); validation (equal); visualization (equal); writing – original draft (lead); writing – review and editing (lead).

## CONFLICT OF INTEREST STATEMENT

There are no competing interests in connection with this manuscript.

## Supporting information


Table S1.

## Data Availability

The data used in this study have been made publicly available at the Institute for Marine and Antarctic Studies (IMAS) Data Portal (https://doi.org/10.25959/70zv‐vs11).

## References

[ece310601-bib-0001] Abraham, E. , & Berkenbusch, K. (2017). Estimated captures of New Zealand fur seal, New Zealand Sea lion, common dolphin, and turtles in New Zealand commercial fisheries, 1995–96 to 2014–15 . New Zealand Aquatic Environment and Biodiversity Report.

[ece310601-bib-0002] Aguilar Soto, N. , Johnson, M. P. , Madsen, P. T. , Diaz, F. , Dominguez, I. , Brito, A. , & Tyack, P. (2008). Cheetahs of the deep sea: Deep foraging sprints in short‐finned pilot whales off Tenerife (Canary Islands). Journal of Animal Ecology, 77, 936–947. 18444999 10.1111/j.1365-2656.2008.01393.x

[ece310601-bib-0003] Augé, A. A. , Chilvers, B. L. , Davis, L. S. , & Moore, A. B. (2011). In the shallow end: Diving behaviour of recolonising female New Zealand Sea lions (*Phocarctos hookeri*) around the Otago peninsula. Canadian Journal of Zoology, 89, 1195–1205.

[ece310601-bib-0004] Augé, A. A. , Chilvers, B. L. , Moore, A. B. , & Davis, L. S. (2011). Foraging behaviour indicates marginal marine habitat for New Zealand Sea lions: Remnant versus recolonising populations. Marine Ecology Progress Series, 432, 247–256.

[ece310601-bib-0005] Augé, A. A. , Chilvers, B. L. , Moore, A. B. , & Davis, L. S. (2014). Importance of studying foraging site fidelity for spatial conservation measures in a mobile predator. Animal Conservation, 17, 61–71.

[ece310601-bib-0060] Baker, C. S. (2019). Conservation status of New Zealand marine mammals, 2019. Laura J. B., Simon C., Rochelle C., Anton Van H., Lundquist D., William R. Wellington, New Zealand. ISBN 978‐1‐988514‐93‐2. OCLC 1143283099.

[ece310601-bib-0006] Bost, C. A. , Cotté, C. , Terray, P. , Barbraud, C. , Bon, C. , Delord, K. , Gimenez, O. , Handrich, Y. , Naito, Y. , Guinet, C. , & Weimerskirch, H. (2015). Large‐scale climatic anomalies affect marine predator foraging behaviour and demography. Nature Communications, 6, 8220. 10.1038/ncomms9220 PMC4639794 26506134

[ece310601-bib-0007] Breed, G. A. , Bowen, W. D. , & Leonard, M. L. (2013). Behavioral signature of intraspecific competition and density dependence in colony‐breeding marine predators. Ecology and Evolution, 3, 3838–3854. 24198943 10.1002/ece3.754 PMC3810878

[ece310601-bib-0008] Butler, P. J. , & Jones, D. R. (1997). Physiology of diving birds and mammals. Physiological Reviews, 77, 837–899. 9234967 10.1152/physrev.1997.77.3.837

[ece310601-bib-0009] Campbell, R. A. , Chilvers, B. L. , Childerhouse, S. , & Gales, N. J. (2006). Conservation management issues and status of the New Zealand (*Phocarctos hookeri*) and Australian (*Neophoca cinerea*) sea lions. In A. W. Trites , S. K. Atkinson , D. Demaster , L. W. Fritz , T. S. Gelatt , L. D. Rea , & K. M. Wynne (Eds.), Sea lions of the world, 2006 Anchorage, Alaska (pp. 455–471). Alaska Sea Grant.

[ece310601-bib-0010] Childerhouse, S. , & Gales, N. (1998). Historical and modern distribution and abundance of the New Zealand Sea lion *Phocarctos hookeri* . New Zealand Journal of Zoology, 25, 1–16.

[ece310601-bib-0011] Childerhouse, S. , Gibbs, N. , Mcalister, G. , Mcconkey, S. , Mcconnell, H. , Mcnally, N. , & Sutherland, D. (2005). Distribution, abundance and growth of New Zealand Sea lion *Phocarctos hookeri* pups on Campbell Island. New Zealand Journal of Marine and Freshwater Research, 39, 889–898.

[ece310601-bib-0012] Childerhouse, S. J. , Dawson, S. M. , Fletcher, D. J. , Slooten, E. , & Chilvers, B. L. (2010). Growth and reproduction of female New Zealand Sea lions. Journal of Mammalogy, 91, 165–176.

[ece310601-bib-0013] Chilvers, B. L. (2008). New Zealand Sea lions *Phocarctos hookeri* and squid trawl fisheries: Bycatch problems and management options. Endangered Species Research, 5, 193–204.

[ece310601-bib-0014] Chilvers, B. L. (2012). Population viability analysis of New Zealand Sea lions, Auckland Islands, New Zealand's sub‐Antarctics: Assessing relative impacts and uncertainty. Polar Biology, 35, 1607–1615.

[ece310601-bib-0015] Chilvers, B. L. (2015). Phocarctos hookeri. The IUCN Red List of Threatened Species. www.iucnredlist.org

[ece310601-bib-0016] Chilvers, B. L. , Amey, J. M. , & Costa, D. P. (2020). Extreme diving of females at the largest colony of New Zealand Sea lions, *Phocarctos hookeri* . Polar Biology, 43, 2031–2042.

[ece310601-bib-0017] Chilvers, B. L. , & Meyer, S. (2017). Conservation needs for the endangered New Zealand Sea lion, *Phocarctos hookeri* . Aquatic Conservation: Marine and Freshwater Ecosystems, 27, 846–855.

[ece310601-bib-0018] Chilvers, B. L. , & Wilkinson, I. S. (2009). Diverse foraging strategies in lactating New Zealand Sea lions. Marine Ecology Progress Series, 378, 299–308.

[ece310601-bib-0019] Chilvers, B. L. , Wilkinson, I. S. , Duignan, P. J. , & Gemmell, N. J. (2006). Diving to extremes: Are New Zealand Sea lions (*Phocarctos hookeri*) pushing their limits in a marginal habitat? Journal of Zoology, 269, 233–240.

[ece310601-bib-0020] Cole, R. G. , Dunn, A. , & Hanchet, S. M. (2013). Review of the time series of input data available for the assessment of southern blue whiting (Micromesistius australis) stocks . New Zealand Fisheries Assessment Report.

[ece310601-bib-0021] Costa, D. P. , & Gales, N. J. (2000). Foraging energetics and diving behavior of lactating New Zealand Sea lions, *Phocarctos hookeri* . Journal of Experimental Biology, 203, 3655–3665. 11060226 10.1242/jeb.203.23.3655

[ece310601-bib-0022] Costa, D. P. , & Gales, N. J. (2003). Energetics of a benthic diver: Seasonal foraging ecology of the Australian sea lion, *Neophoca cinerea* . Ecological Monographs, 73, 27–43.

[ece310601-bib-0023] Costa, D. P. , Kuhn, C. E. , Weise, M. J. , Shaffer, S. A. , & Arnould, J. P. Y. (2004). When does physiology limit the foraging behaviour of freely diving mammals? International Congress Series, 1275, 359–366.

[ece310601-bib-0024] Fowler, S. L. , Costa, D. P. , & Arnould, J. P. Y. (2007). Ontogeny of movements and foraging ranges in the Australian sea lion. Marine Mammal Science, 23, 598–614.

[ece310601-bib-0025] Fowler, S. L. , Costa, D. P. , Arnould, J. P. Y. , Gales, N. J. , & Kuhn, C. E. (2006). Ontogeny of diving behaviour in the Australian sea lion: Trials of adolescence in a late bloomer. Journal of Animal Ecology, 75, 358–367. 16637989 10.1111/j.1365-2656.2006.01055.x

[ece310601-bib-0026] Gales, N. J. , & Mattlin, R. H. (1998). Fast, safe, field‐portable gas anaesthesia for otariids. Marine Mammal Science, 14, 355–361.

[ece310601-bib-0027] Gentry, R. L. (1974). The development of social behavior through play in the Steller Sea lion. American Zoologist, 14, 391–403.

[ece310601-bib-0028] Geschke, K. , & Chilvers, B. L. (2009). Managing big boys: A case study on remote anaesthesia and satellite tracking of adult male New Zealand Sea lions (*Phocarctos hookeri*). Wildlife Research, 36, 666–674.

[ece310601-bib-0029] Hamilton, S. , & Baker, G. B. (2016). Current bycatch levels in Auckland Islands trawl fisheries unlikely to be driving New Zealand Sea lion (*Phocarctos hookeri*) population decline. Aquatic Conservation: Marine and Freshwater Ecosystems, 26, 121–133.

[ece310601-bib-0030] Hamilton, S. , & Baker, G. B. (2019). Population growth of an endangered pinniped—The New Zealand Sea lion (*Phocarctos hookeri*)—Is limited more by high pup mortality than fisheries bycatch. ICES Journal of Marine Science, 76, 1794–1806.

[ece310601-bib-0031] Hazen, E. L. , Abrahms, B. , Brodie, S. , Carroll, G. , Jacox, M. G. , Savoca, M. S. , Scales, K. L. , Sydeman, W. J. , & Bograd, S. J. (2019). Marine top predators as climate and ecosystem sentinels. Frontiers in Ecology and the Environment, 17, 565–574.

[ece310601-bib-0032] Johnson, D. S. (2016). crawl: Fit continuous‐time correlated random walk models to animal movement data . R package version 2.0.

[ece310601-bib-0033] Johnson, D. S. , London, J. M. , Lea, M. A. , & Durban, J. W. (2008). Continuous‐time correlated random walk model for animal telemetry data. Ecology, 89, 1208–1215. 18543615 10.1890/07-1032.1

[ece310601-bib-0034] Kernaléguen, L. , Arnould, J. P. Y. , Guinet, C. , Cazelles, B. , Richard, P. , & Cherel, Y. (2016). Early‐life sexual segregation: Ontogeny of isotopic niche differentiation in the Antarctic fur seal. Scientific Reports, 6, 33211. 27620663 10.1038/srep33211 PMC5020412

[ece310601-bib-0035] Lea, M. A. , Johnson, D. , Melin, S. , Ream, R. , & Gelatt, T. (2010). Diving ontogeny and lunar responses in a highly migratory mammal, the northen fur seal *Callorhinus ursinus* . Marine Ecology Progress Series, 419, 233–247.

[ece310601-bib-0036] Leung, E. S. , Augé, A. A. , Chilvers, B. L. , Moore, A. B. , & Robertson, B. C. (2013). Foraging behaviour of juvenile female New Zealand Sea lions (*Phocarctos hookeri*) in contrasting environments. PLoS ONE, 8, e62728. 23671630 10.1371/journal.pone.0062728 PMC3646001

[ece310601-bib-0037] Leung, E. S. , Chilvers, B. L. , Nakagawa, S. , Moore, A. B. , & Robertson, B. C. (2012). Sexual segregation in juvenile New Zealand Sea lion foraging ranges: Implications for intraspecific competition, population dynamics and conservation. PLoS ONE, 7, e45389. 23028978 10.1371/journal.pone.0045389 PMC3445520

[ece310601-bib-0038] Leung, E. S. , Chilvers, B. L. , Nakagawa, S. , & Robertson, B. C. (2014). Size and experience matter: Diving behaviour of juvenile New Zealand Sea lions (*Phocarctos hookeri)* . Polar Biology, 37, 15–26.

[ece310601-bib-0039] Lindenfors, P. , Tullberg, B. S. , & Biuw, M. (2002). Phylogenetic analyses of sexual selection and sexual size dimorphism in pinnipeds. Behavioral Ecology and Sociobiology, 52, 188–193.

[ece310601-bib-0040] Maloney, A. , Chilvers, B. L. , Haley, M. , Muller, C. G. , Roe, W. , & Debski, I. (2009). Distribution, pup production and mortality of New Zealand Sea lion *Phocarctos hookeri* on Campbell Island/Motu Ihupuku, 2008. New Zealand Journal of Ecology, 33, 97–105.

[ece310601-bib-0041] Maloney, A. , Chilvers, B. L. , Muller, C. G. , & Haley, M. (2012). Increasing pup production of New Zealand Sea lions at Campbell Island/Motu Ihupuku: Can it continue? New Zealand Journal of Zoology, 39, 19–29.

[ece310601-bib-0042] MPI . (2017). National fisheries plan for deepwater and middle‐depth fisheries – Part 1A .

[ece310601-bib-0043] Pinheiro, J. D. B. , Debroy, S. , Sarkar, D. , & R Core Team . (2016). Nlme: Linear and nonlinear mixed effects models . R Package Version 3.1‐128.

[ece310601-bib-0044] Prewitt, J. S. , Freistroffer, D. V. , Schreer, J. F. , Hammill, M. O. , & Burns, J. M. (2010). Postnatal development of muscle biochemistry in nursing harbor seal (*Phoca vitulina*) pups: Limitations to diving behavior? Journal of Comparative Physiology B, 180, 757–766. 10.1007/s00360-010-0448-z 20140678

[ece310601-bib-0045] R Core Team . (2013). R: A language and environment for statistical computing. R Foundation for Statistical Computing.

[ece310601-bib-0046] Raymond, B. , Lea, M. A. , Patterson, T. , Andrews‐Goff, V. , Sharples, R. , Charrassin, J. B. , Cottin, M. , Emmerson, L. , Gales, N. , Gales, R. , Goldworthy, S. D. , Harcourt, R. , Kato, A. , Kirkwood, R. , Lawton, K. , Ropert‐Coudert, Y. , Southwell, C. , Van Den Hoff, Y. , Wienecke, B. , … Hindell, M. (2015). Important marine habitat off East Antarctica revealed by two decades of multi‐species predator tracking. Ecography, 38, 121–129.

[ece310601-bib-0047] Roberts, J. , & Doonan, I. (2016). Quantitative risk assessment of threats to New Zealand Sea lions . New Zealand Aquatic Environment and Biodiversity Report.

[ece310601-bib-0048] Robertson, B. C. , & Chilvers, B. L. (2011). The population decline of the New Zealand Sea lion *Phocarctos hookeri*: A review of possible causes. Mammal Review, 41, 253–275.

[ece310601-bib-0049] Roman, J. , Estes, J. A. , Morissette, L. , Smith, C. , Costa, D. , Mccarthy, J. , Nation, J. , Nicol, S. , Pershing, A. , & Smetacek, V. (2014). Whales as marine ecosystem engineers. Frontiers in Ecology and the Environment, 12, 377–385.

[ece310601-bib-0050] Sterling, J. T. , Springer, A. M. , Iverson, S. J. , Johnson, S. P. , Pelland, N. A. , Johnson, D. S. , Lea, M.‐ A. , & Bond, N. A. (2014). The sun, moon, wind, and biological imperative ‐ shaping contrasting wintertime migration and foraging strategies of adult male and female northern fur seals (*Callorhinus ursinus*). PLoS ONE, 9, 1–21. 10.1371/journal.pone.0093068 PMC3983057 24722344

[ece310601-bib-0051] Thompson, F. N. , & Abraham, E. R. (2010). Estimation of the capture of New Zealand Sea lions (Phocarctos hookeri) in trawl fisheries, from 1995–96 to 2008–09 . In Fisheries, M. O. (Ed.) New Zealand aquatic environment and biodiversity technical report.

[ece310601-bib-0052] Thompson, F. N. , Berkenbusch, K. , & Beritzhoff‐Law, M. (2015). Reported New Zealand Sea lion (Phocarctos hookeri) captures in commercial trawl Fisheries, 1991–92 to 2012–13 . In Industries, M. F. P. (Ed.).

[ece310601-bib-0053] Trites, A. W. , Atkinson, S. K. , Demaster, D. P. , Fritz, L. W. , Gelatt, T. S. , Rea, L. D. , & Wuynne, K. M. (Eds.). (2006). Sea lions of the world. 2004. Alaska Sea Grant.

[ece310601-bib-0054] Villegas‐Amtmann, S. , Costa, D. P. , Tremblay, Y. , Salazar, S. , & Aurioles‐Gamboa, D. (2008). Multiple foraging strategies in a marine apex predator, the Galapagos Sea lion *Zalophus wollebaeki* . Marine Ecology Progress Series, 363, 299–309.

[ece310601-bib-0055] Vincent, C. , Mcconnell, B. J. , Ridoux, V. , & Fedak, M. A. (2002). Assessment of Argos location accuracy from satellite tags deployed on captive gray seals. Marine Mammal Science, 18, 156–166.

[ece310601-bib-0056] Viviant, M. , Monestiez, P. , & Guinet, C. (2014). Can we predict foraging success in a marine predator from dive patterns only? Validation with prey capture attempt data. PLoS ONE, 9, e88503. 24603534 10.1371/journal.pone.0088503 PMC3945944

[ece310601-bib-0057] Weise, M. J. , & Costa, D. P. (2007). Total body oxygen stores and physiological diving capacity of California Sea lions as a function of sex and age. Journal of Experimental Biology, 210, 278–289. 17210964 10.1242/jeb.02643

[ece310601-bib-0058] Wirsing, A. J. , Heithaus, M. R. , Frid, A. , & Dill, L. M. (2008). Seascapes of fear: Evaluating sublethal predator effects experienced and generated by marine mammals. Marine Mammal Science, 24, 1–15.

[ece310601-bib-0059] Womble, J. N. , Horning, M. , Lea, M. A. , & Rehberg, J. (2013). Diving into the analysis of time‐depth recorder and behavioural data records: A workshop summary. Deep‐Sea Research. Part 2: Topical Studies in Oceanography, 88–89, 61–64.

