# Supplementary material for: The benthic‐pelagic continuum: Age class and sex differences in the use of the vertical dimension by a rare pinniped
Source: Ecol Evol. 2023 Nov 2;13(11):e10601. doi: 10.1002/ece3.10601 (PMC10622853; doi:10.1002/ece3.10601)
Supplement: Supplementary file 1 — Table S1. [file ECE3-13-e10601-s001.docx]

**SI Table 1.** Dive behaviour data compiled for published studies from the Auckland Islands and the Otago Peninsula.

| Site | Age class | Sex | Depth (m) | Duration (min.) | Citation |
| --- | --- | --- | --- | --- | --- |
| Otago Peninsula | Adult | F | 15.7 | 1.4 | 1 |
|  | Adult | F | 17.7 | 2.1 | Dives ≥3m |
|  | Adult | F | 30.1 | 2.1 |  |
|  | Adult | F | 13.9 | 1.6 |  |
|  | Adult | F | 39.3 | 2.2 |  |
|  | Juvenile | F | 16.0 | 1.7 |  |
|  | Juvenile | F | 30.2 | 1.5 |  |
|  | Juvenile | F | 8.8 | 1.3 |  |
|  | Juvenile | F | 15.8 | 1.7 |  |
|  | Juvenile | F | 42.5 | 2.1 |  |
|  | Juvenile | F | 15.3 | 1.8 |  |
|  | Juvenile | F | 26.2 | 1.6 |  |
|  | Juvenile | F | 20.1 | 1.7 |  |
| Auckland Islands | Adult | F | 140 | 3 | 2 |
|  | Adult | F | 92 | 3 | Dives >6m |
|  | Adult | F | 111 | 4 |  |
|  | Adult | F | 69 | 2.6 |  |
|  | Adult | F | 99 | 3.3 |  |
|  | Adult | F | 97 | 2.6 |  |
|  | Adult | F | 180 | 4.1 |  |
|  | Adult | F | 165 | 3.6 |  |
|  | Adult | F | 155 | 3.4 |  |
|  | Adult | F | 135 | 3.8 |  |
|  | Adult | F | 153.3 | 1.8 |  |
|  | Adult | F | 81.7 | 1.5 |  |
|  | Adult | F | 112.4 | 1.3 | 3 |
|  | Adult | F | 79 | 2.2 | Dives ≥6m |
|  | Adult | F | 186.9 | 2.7 |  |
|  | Adult | F | 136.6 | 1.5 |  |
|  | Adult | F | 103.6 | 1.7 |  |
|  | Adult | F | 102.5 | 1.9 |  |
|  | Adult | F | 151.7 | 1.8 |  |
|  | Adult | F | 139.3 | 2.5 |  |
|  | Adult | F | 94.2 | 1.2 |  |
|  | Adult | F | 125.8 | 1.8 |  |
|  | Adult | F | 103.1 | 1.8 |  |
|  | Adult | F | 175.3 | 2.4 |  |
|  | Adult | F | 142.4 | 4.42 | 4,5 |
|  | Adult | F | 178.9 | 4.97 | Dives ≥6m |
|  | Adult | F | 147.3 | 3.64 |  |
|  | Adult | F | 114.1 | 4.29 |  |
|  | Adult | F | 134.3 | 4.48 |  |
|  | Adult | F | 169.8 | 3.89 |  |
|  | Adult | F | 119.5 | 4.01 |  |
|  | Adult | F | 109.7 | 4.39 |  |
| Auckland Islands | Adult | F | 128.4 | 3.56 |  |
|  | Adult | F | 119.2 | 4.18 |  |
|  | Adult | F | 135.4 | 3.51 |  |
|  | Adult | F | 133.9 | 4.11 |  |
|  | Adult | F | 130.9 | 3.59 |  |
|  | Adult | F | 129.5 | 3.45 |  |
|  | Adult | F | 143 | 4.14 |  |
|  | Adult | F | 94.6 | 3.92 |  |
|  | Adult | F | 97.6 | 3.37 |  |
|  | Adult | F | 101.4 | 3.96 |  |
|  | Juvenile | F | 24 | 1.8 | 6 |
|  | Juvenile | F | 40.1 | 2.5 | Dives ≥6m |
|  | Juvenile | M | 43.1 | 2 |  |
|  | Juvenile | M | 38.7 | 2.3 |  |
|  | Juvenile | M | 35 | 2.1 |  |
|  | Juvenile | M | 19.3 | 1.4 |  |
|  | Juvenile | F | 98 | 3.19 | 7 |
|  | Juvenile | F | 100.7 | 3.12 | Dives ≥6m |
|  | Juvenile | M | 74 | 2.99 |  |
|  | Juvenile | M | 149.2 | 4.58 |  |
|  | Juvenile | F | 122.3 | 3.73 |  |

1 (Augé et al. 2011), 2 (Costa and Gales 2000), 3 (Gales and Mattlin 1997) 4,5 (Chilvers et al. 2006) (Chilvers and Wilkinson 2009) 6 (Leung et al. 2013) 7 (Leung et al. 2014)

**Reference**

Augé, A. A., B. L. Chilvers, et al. 2011. In the shallow end: diving behaviour of recolonising female New Zealand sea lions (*Phocarctos hookeri*) around the Otago Peninsula. *Canadian Journal of Zoology* **89**:1195-1205.

Chilvers, B. L., and I. S. Wilkinson. 2009. Diverse foraging strategies in lactating New Zealand sea lions. *Marine Ecology Progress Series* **378**:299-308.

Chilvers, B. L., I. S. Wilkinson, et al. 2006. Diving to extremes: are New Zealand sea lions (*Phocarctos hookeri*) pushing their limits in a marginal habitat? *Journal of Zoology* **269**:233-240.

Costa, D. P., and N. J. Gales. 2000. Foraging energetics and diving behavior of lactating New Zealand sea lions, *Phocarctos hookeri*. Journal of Experimental Biology **203**:3655-3665.

Gales, N. J., and R. H. Mattlin. 1997. Summer diving behaviour of lactating New Zealand sea lions, *Phocarctos hookeri. Canadian Journal of Zoology* **75**:1695-1706.

Leung, E. S., B. L. Chilvers, et al. 2013. Mass and bathymetry influences on the foraging behaviour of dependent yearling New Zealand sea lions (*Phocarctos hookeri*). *New Zealand Journal of Marine and Freshwater Research* **47**:38-50.

Leung, E. S., B. L. Chilvers, et al. 2014. Size and experience matter: diving behaviour of juvenile New Zealand sea lions (*Phocarctos hookeri)*. *Polar Biology* **37**:15-26.
